# Identification and Molecular Mechanisms of Key Nucleotides Causing Attenuation in Pathogenicity of Dahlia Isolate of Potato Spindle Tuber Viroid

**DOI:** 10.3390/ijms21197352

**Published:** 2020-10-05

**Authors:** Shoya Kitabayashi, Daiki Tsushima, Charith Raj Adkar-Purushothama, Teruo Sano

**Affiliations:** 1 Faculty of Agriculture and Life Science, Hirosaki University, Hirosaki 036-8561, Japan; kitabayashis640@affrc.go.jp; 2 Department of Neuropsychiatry, Graduate School of Medicine, Hirosaki University, 5 Zaifu-cho, Hirosaki, Aomori 036-8562, Japan; tsushima-d@hirosaki-u.ac.jp; 3 RNA Group/Groupe ARN, Département de Biochimie, Faculté de Médecine des Sciences de la Santé, Pavillon de Recherche Appliquée au Cancer, Université de Sherbrooke, 3201 rue Jean Mignault, Sherbrooke, QC J1E 4K8, Canada

**Keywords:** PSTVd, viroid pathogenicity, PSTVd-D, mutation

## Abstract

While the potato spindle tuber viroid (PSTVd) variant, PSTVd-Dahlia (PSTVd-D or PSTVd-D_wt_) induces very mild symptoms in tomato cultivar ‘Rutgers’, PSTVd-Intermediate (PSTVd-I or PSTVd-I_wt_) induces severe symptoms. These two variants differ by nine nucleotides, of which six mutations are located in the terminal left (TL) to the pathogenicity (P) domains. To evaluate the importance of mutations located in the TL to the P domains, ten types of point mutants were created by swapping the nucleotides between the two viroid variants. Bioassay in tomato plants demonstrated that two mutants created on PSTVd-I_wt_ at positions 42 and 64 resulted in symptom attenuation. Phenotypic and RT-qPCR analysis revealed that mutation at position 42 of PSTVd-I_wt_ significantly reduced disease severity and accumulation of the viroid, whereas mutation at position 64 showed a significant reduction in stunting when compared to the PSTVd-I_wt_ infected plant. RT-qPCR analysis on pathogenesis-related protein 1b1 and chalcone synthase genes showed a direct correlation with symptom severity whereas the expansin genes were down-regulated irrespective of the symptom severity. These results indicate that the nucleotides at positions 42 and 64 are in concert with the ones at positions 43, 310, and 311/312, which determines the slower and stable accumulation of PSTVd-D without eliciting excessive host defense responses thus contributing in the attenuation of disease symptom.

## 1. Introduction

Viroids are non-coding RNA pathogens that do not encode any protein. They consist of a circular single-stranded RNA, about 250–434 nucleotides in length [1,2,3]. After invading host cells through wounds, viroids translocate to either nucleus or chloroplast depending on the viroid species, then multiply through “rolling circle replication”, completely depending on the host transcription machinery, and move to adjacent cells through plasmodesmata and spread systemically via the phloem transport system [4,5,6]. Currently, more than 32 viroids have been reported, and they are classified into two families and eight genera based on the mode of replication, intracellular localization, nucleotide sequence homology, and the presence or absence of conserved regions and motifs [7,8].

Potato spindle tuber viroid (PSTVd) is the first viroid discovered as a causal agent of spindle tuber disease of potato in North America [9,10]. PSTVd mainly infects solanaceous plants and causes a variety of symptoms such as dwarfing, epinasty, leaf malformation, chlorosis, vein necrosis, flower spotting, and cracking of tuber or discoloration of fruit on sensitive host varieties [11]. Meanwhile, a majority of flowers and ornamental plants belonging to the *Solanaceae* and *Asteraceae* families are symptomless carriers of the viroid [12]. The complete nucleotide sequence of the PSTVd-type isolate, namely, “Intermediate” strain (PSTVd-I or PSTVd-I_wt_), has been determined. It is a circular single-stranded RNA composed of 359 nucleotides and was found to form a highly intramolecularly base-paired rod-shaped stem-loop secondary structure [13]. The circular RNA was found to be composed of five functional/structural domains named Terminal left (TL), Pathogenicity (P), Central (C), Variable (V), and Terminal right (TR) [14]. So far, more than 300 PSTVd strains or variants have been reported, and numerous mutations have been identified throughout the genome; however, many of them are concentrated in the P and V domains [15,16,17,18,19,20,21,22,23,24]. Analysis of mutations found in PSTVd isolates with different pathogenicity to tomato (*Solanum lycopersicum*) strongly supported the premise that nucleotide sequences or structures in the P domain play a major role in determining pathogenicity. For example, changes in nucleotides in the P domain named Virulence Modulating Region (VMR; a nucleotide sequence located around nucleotide positions 43–59) and Pre-Melting 1 region (PM1; at nucleotides positions 50–59) have been suggested to induce changes in the thermodynamic stability of the local secondary structure and can cause differences in virulence through interaction with unidentified host factor(s) [15,25,26,27,28,29]. The introduction of random single-nucleotide mutations in the V and P domains resulted in a drastic decrease in accumulation and loss of replication ability [30,31]. In addition, the C domain was also shown to play a critical role in pathogenicity. For instance substituting the nucleotide at position 257 in the lower strand of the Central Conserved Region (CCR) in the C domain of PSTVd-I_wt_ from uracil (U) to adenine (A), changed the mutant into a lethal symptom expressing type [32], and substitution of the nucleotide at 259 in the tetra-loop E motif in the C domain of PSTVd-I_wt_ from U to cytosine (C) changed the mutants that could infect tobacco (*Nicotiana tabacum*) more efficiently [33].

On the other hand, viroids are strong RNA silencing inducers, and viroid-infected plants accumulate large amounts of viroid-specific small RNA (vd-sRNA) [34,35,36]. It has been suggested that vd-sRNA may be involved in viroid pathogenicity by targeting and inhibiting host gene expression with highly complementary sequences [37,38,39,40]. A vd-sRNA produced through infection of a specific mutant of peach latent mosaic viroid (PLMVd) was found for the first time to incite a post-transcriptional suppression of mRNA encoding chloroplastic heat shock protein 90, causing peach calico disease [41]. A vd-sRNA generated from the VMR of PSTVd was then shown to silence the callose synthase genes of tomato plants, and it was observed that suppression was more pronounced in plants infected with a severe strain than those infected with a mild strain [42]. More recently, vd-sRNAs derived from the VMR of PSTVd were reported to have silenced a transcription factor encoding gene *StTCP23* in potato, leading to the development of disease symptoms [43]. Moreover, genes reported containing sequences that could be targeted by vd-sRNAs include tomato FRIGIDA-*like* protein 3 (*FRL3*), *chloride channel protein CLCb*-like (*clcB*), *ribosomal protein S3a*-*like* (*RPS3A*), *serine-threonine protein kinases*, *WD40-repeat protein* (*WD40*), and *soluble inorganic pyrophosphatase* of tobacco plants [44,45,46,47,48,49,50].

However, systemic symptoms such as dwarfing, leaf malformation, yellowing, and necrosis caused by viroid infection cannot be exclusively attributed to the action of vd-sRNAs. Therefore, it is believed that there are several causes for viroid induced systemic symptoms [51,52]. Recent technological advances in the analysis of gene expressions, such as microarrays and RNA sequencing (RNA-Seq), have enabled researchers to analyze the comprehensive transcriptomic changes in various plants infected with viroids. In the case of PSTVd-infected tomato plants, the infection caused genome-wide alteration of alternative splicing of the gene encoding proteins, enhanced regulation of gene expression by host microRNA, and improved induction of RNA interference by phased siRNA (phasiRNA) [53]. Furthermore, it was shown in various viroid–host combinations that genes involved in plant innate immunity, mainly calcium-dependent protein kinase and mitogen-activated protein kinase cascade or genes involved in hypersensitive response, cell wall enhancement, and hormone signaling were activated by viroid infection. These observations also suggested that the expression of a large number of genes, such as those involved in defense responses, hormone metabolisms, signal transduction, photosynthesis, chloroplast, cell wall, and regulation of RNA expression are affected by viroid infection, leading to the development of disease symptoms [53,54,55,56,57,58,59,60].

Furthermore, it was discovered that the flower plant dahlia (*Dahlia pinnata*) was latently infected with PSTVd [61]. The dahlia isolate of PSTVd (PSTVd-D of PSTVd-D_wt_) induces mild symptoms on a PSTVd-sensitive tomato cultivar ‘Rutgers’ when compared to the reference strain, PSTVd-I (or PSTVd-I_wt_). The nucleotide sequence homology between these isolates was approximately 97%. Specifically, the two isolates, PSTVd-I_wt_ and PSTVd-D_wt_ differ by the following nine mutations at nucleotide positions 42 (C to U) and 43 (U to C) in the TL domain; 64 (U insertion), 310 (A to C), and 311/312 (UU insertion) in the P domain;119 (A deletion) and 126 (A to C) in the V domain; and 201 (G to U) in the TR domain, referring to the number of PSTVd-D_wt_ [61,62]. In the present study, the six mutations in the TL and P domains of the PSTVd were considered to understand the molecular mechanism in symptom induction on tomato plants.

## 2. Results

### 2.1. Infectivity and Genetic Stability of PSTVd Mutants

**Figure 1:**
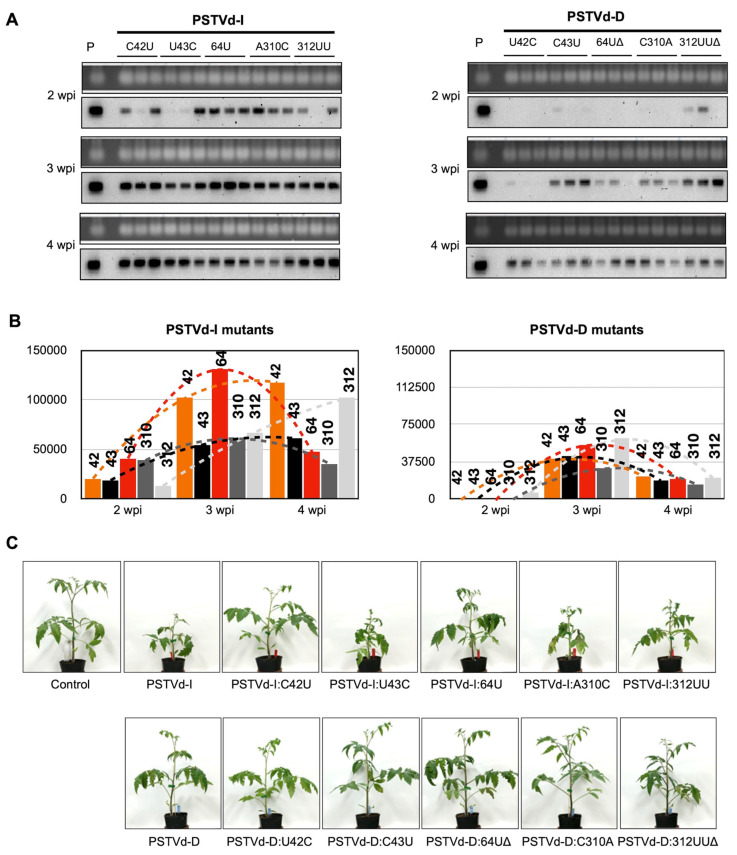
Comparison of accumulation of viroid RNA and severity of symptoms of tomato plants infected with two PSTVd variants and its mutatns. Total RNA was extracted from tomato plants infected with in vitro transcripts of 2 variants of PSTVd and its mutants at 2-, 3- and 4-wpi were used for (**A**) Northern blot hybridization using digoxigenin (DIG)-labelled PSTVd specific probes to analyze the viroid RNA accumulation. P denotes positive control from PSTVd RNA and Control indicates a mock-inoculated tomato plant. (**B**) Accumulation of viroid RNA was evaluated by quantifying signals obtained in Northern blot hybridization. Number on the bars represents the nucleotide position that has been mutated. The changes in the viroid RNA accumulation between the time points are shown with dotted lines. (**C**) At 4-wpi, PSTVd mutants caused an array of symptoms on tomato plants.

To evaluate the infectivity of PSTVd mutants constructed in this study, tomato plants were inoculated with in vitro transcripts of dimeric PSTVd mutants. Northern blot hybridization analysis of total RNA extracted at 2-, 3- and 4-wpi showed that all 10 single mutants of PSTVd had the capacity to infect (Figure 1A). At 2-wpi, at least one plant inoculated with each PSTVd-I mutant revealed the presence of viroid RNA, in contrast, only one of the PSTVd-D mutants, PSTVd-D:312UUΔ was detectable. At 3-wpi, although all mutants were infective, two of the PSTVd-D mutants, PSTVd-D:U42C and PSTVd-D:64UΔ, exhibited delayed accumulation.

Hybridization signals obtained for 2-, 3-, and 4-wpi were quantified using QuantityOne software. As presented in Figure 1B, the accumulation of PSTVd-D mutants is 50% less than that of PSTVd-I mutants at any point of infection. It is interesting to note that PSTVd-I:64U and PSTVd-I:A310C showed the highest accumulation at 3-wpi followed by a decrease in concentration, whereas other mutants (PSTVd-I:C42U, PSTVd-I:U43C and PSTVd-I:312UU) demonstrated an increase in accumulation from 2- to 4-wpi. All the PSTVd-D mutants accumulated to the maximum at 3-wpi before showing a decline in 4-wpi. The visual observation for tomato phenotypes induced by PSTVd variants and its mutants at 4-wpi indicated that the PSTVd-D derived mutants induced mild symptoms and accumulated slowly when compared to PSTVd-I_wt_ infected plants (Figure 1C). Although all the PSTVd-I derived mutants inoculated tomato plants exhibited severe dwarfing and leaf malformation symptoms and accumulated faster, their symptom severity and rate of accumulation were less than that of the PSTVd-I_wt_. Interestingly, the PSTVd-I:C42U and PSTVd-I:64U inoculated plants showed very mild leaf curl symptom on the top of the plant at 4-wpi, which is comparable to the symptoms observed in PSTVd-D_wt_ infected tomato plants. On the other hand, PSTVd-D:U42C showed a slight increase in symptoms compared to PSTVd-D_wt_, although this mutant retained the introduced U42C mutation but was accompanied by a high C to U transition at the adjacent nucleotide 43 (Table 1). These results suggest that the mutations introduced at nucleotide positions 42 and 64 are important for the attenuation of PSTVd.

The sequence data of 10 to 14 cDNA clones per progeny isolated at 4-wpi samples were used to verify the genetic stability of the mutants. Data analysis revealed that three PSTVd-I mutants (PSTVd-I:C42U, PSTVd-I:64U, and PSTVd-I:A310C) and two PSTVd-D mutants (PSTVd-D:C43U and PSTVd-D:312UUΔ) replicated stably. In contrast, two PSTVd-I mutants and three PSTVd-D mutants were not stable during the infectivity assay. Specifically, two of 13 progenies recovered from PSTVd-I:U43C infected plants reverted to PSTVd-I_wt_ type (Table 1). PSTVd-I:312UU was also unstable and 10 of 12 progenies sequenced lost a U from 312UU inserted (Table 1). Although the introduced mutation was maintained stably in three PSTVd-D mutants; i.e., PSTVd-D:U42C, PSTVd-D:64Δ, and PSTVd-D:C310A, they showed covariations in the other nucleotide(s) at C43U (10 of 11 cDNA clones), 311CΔ (5 of 12), and 312UΔ (5 of 10), respectively (Table 1).

Since reversion or covariation was observed in some of the cDNA clones analyzed from the PSTVd transcripts infected plants, the second passage of infectivity assay was conducted with native viroid RNAs extracted from the in vitro transcript-infected tomato plants, to confirm the stability of the mutants. The second passage of infectivity assay showed that all the mutants accumulated more rapidly than in the case of in vitro transcripts infection assay. Northern blot assays on the total RNA extracted for the detection of PSTVd RNA revealed that all the PSTVd-I mutants were detectable at 3-wpi. Although one out of three plants inoculated with PSTVd-D_wt_ showed the presence of PSTVd at 3-wpi, almost all the plants inoculated with PSTVd-D mutants exhibited the presence of viroid RNA indicating that all five-point mutations on PSTVd-D_wt_ did not affect its accumulation (Figure S1).

Sequence analysis of progenies recovered from LMW-RNA infected plants revealed that all five mutants which were stable in the first infectivity assay (PSTVd-I:C42U, PSTVd-I:64U, PSTVd-I:A310C, PSTVd-D:C43U and PSTVd-D:312UUΔ) remained stable in the second passage indicating that these mutations are indeed stable (Table 1). Conversely, the ratio of revertant or covariation of five unstable mutants observed in the first infectivity assay (PSTVd-I:U43C, PSTVd-I:312UU, PSTVd-D:U42C, PSTVd-D:64Δ and PSTVd-D:C310A) were further increased in the second passage. All the clones obtained in the second passage of unstable mutant infected plants showed 100% reversion to the wild type or covariation. 

### 2.2. Mutation at Positions 42 and 64 Attenuated Symptoms

To elucidate the role of nucleotides 42 and 64 of PSTVd in attenuation, accumulation, and symptom induction in the host plant, a comparative bioassay was performed using PSTVd-I:C42U, PSTVd-I:64U, PSTVd-I:C42U/64U, PSTVd-I_wt_, and PSTVd-D_wt_ in tomato plants. Before initiating the comparative assay, the infectivity and genetic stability of PSTVd-I:C42U/64U were analyzed as before. Although RT-PCR assays on total RNA extracted at 4-wpi from the tomato plants inoculated with transcripts of PSTVd-I:C42U/64U revealed the presence of viroid RNA in all the three inoculated tomato plants, the Northern blot hybridization showed presence of viroid RNA only in one plant (Figure S2). This discrepancy might be attributed to the difference in sensitivity between the two assays. Sequence analysis of five selected cDNA clones of the progeny confirmed the stability of double mutants, indicating that PSTVd-I:C42U/64U is a stable mutant and can be used in subsequent comparative experiments.

**Figure 2:**
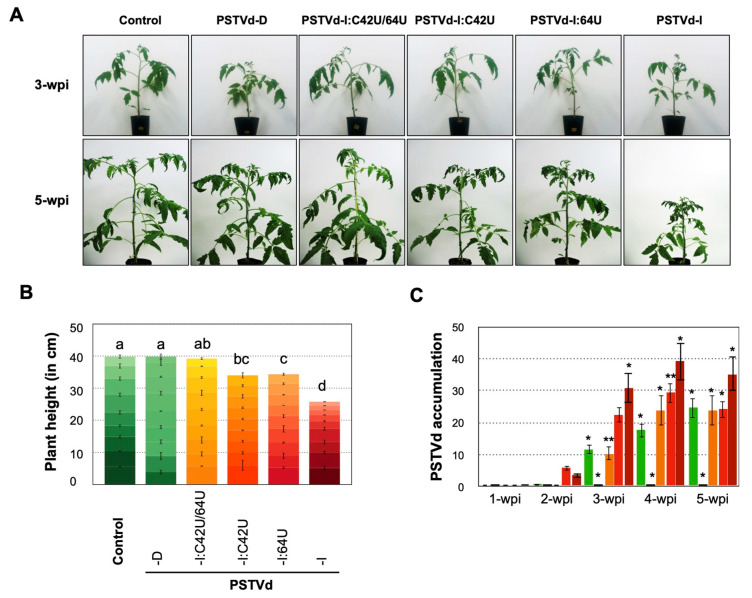
Mutation at nucleotide positions 42 and 64 of PSTVd-I attenuated symptom in tomato plants. Tomato plants inoculated with low molecular weight (LMW)-RNA of PSTVd-D_wt_, PSTVd-I_wt_, and mutants of PSTVd-I (PSTVd-I:C42U, PSTVd-I:64U and, PSTVd-I:C42U/64U) to examine the role of specific nucleotides in symptom attenuation. (**A**) Plants inoculated with LMW-RNA of PSTVd-I_wt_ exhibited leaf malformation at 3-wpi and severe dwarfing symptoms at 5-wpi whereas LMW-RNA of PSTVd-D inoculated plants resembled that of mock-inoculated control even at 5 wpi. Although plants inoculated with LMW-RNA of PSTVd-I:C42U and PSTVd-I:64U exhibited mild leaf malformation at 5-wpi, none of the plants inoculated with LMW-RNA of PSTVd-I:C42U/64U show any visible symptoms. (**B**) A schematic diagram of the comparison of the 9 internodes and accumulated height of the mock-inoculated control plants, and those infected with PSTVd-D_wt_, PSTVd-I:C42U/64U, PSTVd-I:64U, PSTVd-I:C42U, and PSTVd-I_wt_ at 5-wpi. Different letters (a to d) on the top of the bars indicate statistical significance with a *p*-value of 0.05 (*t*-test). Mean values are based on three replicates. (**C**) Comparative analysis of the accumulation of viroid RNAs in infected tomato plants in time course (1- to 5-wpi) by Northern blot hybridization. Accumulation of viroid RNA was calculated by measuring the band intensity in ChemiDoc-XRS by Quantity One software and normalized by the mean value of PSTVd-I_wt_ at 2-wpi as 1.0. A *t*-test was performed with a *p*-value of 0.05. * and ** on the top of the bars represent a significant difference of <0.05 and <0.01, respectively. Mean values are based on three biological and three technical replicates. In the figure, green bars indicate PSTVd-D_wt_ inoculated plants, yellow-colored bars indicate PSTVd-I:C42U/64U inoculated plants, orange-colored bars indicate PSTVd-I:C42U inoculated plants, red bars indicate PSTVd-I:64U inoculated plants, dark red colored bars indicate PSTVd-I_wt_ inoculated plants, respectively.

For the comparative infection assay, a group of nine tomato seedlings were infected with LMW-RNA of the mutants and PSTVd-I_wt_ and PSTVd-D_wt_ as control. The plants inoculated with PSTVd-I_wt_ exhibited leaf malformation at 3-wpi and severe dwarfing symptoms 4- to 5-wpi. On the other hand, PSTVd-D_wt_ did not show any disease symptoms even at 5-wpi. The plants inoculated with PSTVd-I:C42U and PSTVd-I:64U exhibited mild leaf malformation at 5-wpi. However, none of the plants inoculated with PSTVd-I:C42U/64U showed visible disease symptoms even at 5-wpi (Figure 2A). Plants inoculated with PSTVd-I and PSTVd-I:64U showed severe vein necrosis on some fully expanded leaves at 5-wpi (Figure S3). At 5-wpi, the internode length and the total height of the plants inoculated with PSTVd-D_wt_ or PSTVd-I:C42U/64U was evidence enough to conclude that plants were devoid of visible symptoms. The plants inoculated with PSTVd-I:C42U and PSTVd-I:64U were 34.0 cm and 34.3 cm in height, which are significantly shorter than the mock-inoculated control plants (39.8 cm), whereas those infected with PSTVd-I_wt_ (25.7 cm) were much shorter than all other PSTVd inoculated and mock-inoculated control plants (Figure 2B). These results indicated the direct involvement of nucleotides at positions 42 and 64 in attenuating PSTVd-induced symptoms.

To evaluate the effect of mutations on the PSTVd on accumulation, total RNA extracted from the plants inoculated with PSTVd mutations at 1- to 5-wpi were examined by Northern blot hybridization (Figure 2C). Results showed that the PSTVd-I_wt_ and PSTVd-I:64U were detected at 2-wpi followed by PSTVd-D_wt_ and PSTVd-I:C42U at 3-wpi. However, PSTVd-I:C42U/64U was hardly detectable even at 5-wpi. Quantification of such detected bands after normalization revealed that, though PSTVd-I:64U accumulated slightly higher than PSTVd-I_wt_ at 2-wpi, the latter was preceded in later weeks. Detectable difference in the accumulation of PSTVd-D_wt_ and PSTVd-I:C42U were not observed and were significantly lower than those of PSTVd-I_wt_ and PSTVd-I:C64U. These results were supported by RT-qPCR analysis (Figure S4). Taken together, these data demonstrated that: (i) the mutants with U at nucleotide 42 were slower to accumulate than those with C, and (ii) the mutation at nucleotide 64 was found to be less important in symptom attenuation than that at position 42.

### 2.3. Impact of Mutation on PSTVd Secondary Structure

**Figure 3:**
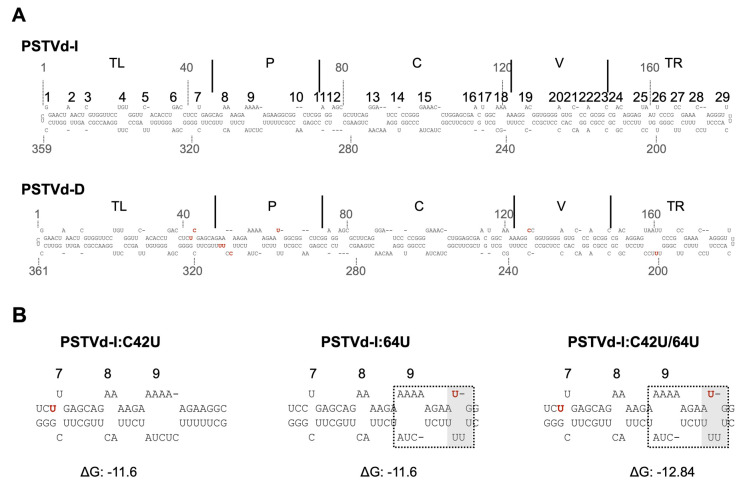
In silico secondary structure prediction of PSTVd mutants. Secondary structure of PSTVd-I_wt_, PSTVd-D_wt_, PSTVd-I:C42U, PSTVd-I:64U, and PSTVd-I:C42U/64U are predicted in silico using mfold webserver. (**A**) Full-length structure of PSTVd-I_wt_ and PSTVd-D_wt_ are presented. The structural/functional domains of PSTVd: Terminal left (TL), Pathogenicity (P), Central (C), Variable (V), and Terminal right (TR), are delimited by the vertical solid lines and are named accordingly. The 29 loops are numbered from left to right on PSTVd-I. The red colored nucleotides on the PSTVd-D_wt_ structure represents the nucleotides that are different from PSTVd-I_wt_. (**B**) Enlarged structure of PSTVd-I at the mutated region. The red color nucleotide indicates the mutated nucleotide. Structural changes observed against the PSTVd-I_wt_ at the site of mutation are shown by a grey box. The dotted box indicates the structural deviation observed in mutants compared to the secondary structure of PSTVd-I. An online tool, PairFold, was used to predict the minimum secondary structure free energy of pairs of RNA sequences.

It has been suggested that the secondary structure of the VM region in the P domain of PSTVd may play an important role in virulence against tomato [15,25,26,27,28,29,63]. Therefore, the effects of mutations at nucleotides 42 and 64 on the secondary structure of the P domain were analyzed by in silico thermodynamic folding (Figure 3). Although mutation at nucleotide 42 did not affect the secondary structure of the PSTVd-I_wt_, it has changed Watson–Crick base pairing (C-G) to a wobble base pairing (U:G). It is worth noting that the previous *in solution* structure analysis of PSTVd-D_wt_ revealed an increase in the size of loop 7 at this position instead of forming wobble base-pairing as predicted in silico [42]. The insertion of U at nucleotide position 64 of PSTVd-I_wt_ resulted in the formation of a new loop between loop 9 and 10. The formation of a new loop resulted in a decrease in the size of loop 9 giving the structural similarity with that of PSTVd-D_wt_. The double mutant, PSTVd-I:C42U/64U, resulted in a hybrid of two mutant structures as shown in Figure 3. Due to these mutations, PSTVd-I_wt_ showed structural resemblance to PSTVd-D_wt_ at mutated points. Comparing the overall structure of PSTVd-D_wt_ with PSTVd-I_wt_ and its mutants, the seventh stem of PSTVd-D_wt_ was extended by 2 bp. The minimum free energy of the secondary structure of the VM region was lower (−12.8 kcal/mol) only in PSTVd-D_wt_ and all the same (−11.1 kcal/mol) in the other four regardless of their pathogenicity. These differences in the secondary structure of PSTVd-D_wt_ maybe attributed to the C-to-A transversion of nucleotide 310 in the lower chain of the P domain and the insertion of UU in position 312.

### 2.4. Identification of Three Tomato Genes Whose Expression Levels Fluctuate to Virulence of PSTVd Mutants

Changes in host transcriptome caused by viroid infection have been analyzed in various viroid–host combinations [53,54,55,56,57,58,59,60]. Infected plants are characterized by the fluctuations in several observables such as expressions of genes involved in plant innate immunity, defense responses, signal transduction and hormone metabolism. The fluctuations lead to the development of disease symptoms. The pathogenicity-related protein 1B1 (*PR1b1*), *TCHS2* and *expansin* (*slEXPA*) genes [32,53,59,64,65,66], among others, can be clearly associated with viroid disease symptoms. Samples collected from PSTVd mutants infected plants were analyzed to determine the correlation between the expression of three previously identified genes and the severity of disease symptoms.

**Figure 4:**
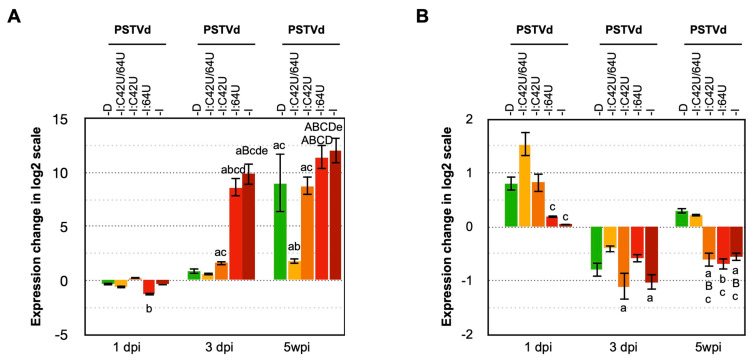
Effect of PSTVd infection on the expression of *PR1b1* and *TCHS2*. Total RNA extracted from tomato plants inoculated with LMW-RNA of PSTVd-D_wt_, PSTVd-I_wt_ and mutants of PSTVd-I (PSTVd-I:C42U/64U, PSTVd-I:C42U, and PSTVd-I:64U) at 1-, 3- and 5-wpi were assayed by RT-qPCR to analyze the expression level of (**A**) *PR1b1* and (**B**) *TCHS2* mRNAs. The vertical axis indicates the relative expression level (Log2) of mRNAs. The data were normalized by the mean value of the mock-inoculated control at 1-wpi as 1.0. A *t*-test was performed with a *p*-value of 0.05. The plants that showed a difference < 0.05 and <0.01 are indicated by lower and capital letters on the top of the bars, respectively. In the figure, significantly difference against the mock-inoculated control plant, PSTVd-D_wt_, PSTVd-I:C42U/64U, PSTVd-I:C42U, and PSTVd-I:64U is represented as a or A, b or B, c or C, d or D, and e or E, respectively. Mean values are based on three biological and three technical replicates.

The RT-qPCR analysis revealed that the plants infected with PSTVd-I_wt_ and PSTVd-I:64U showed increased expression of *PR1b1* mRNA as early as 3-wpi, whereas PSTVd-D_wt_ and PSTVd-I:C42U plants eventually exhibited an increase in *PR1b1* mRNA at 5-wpi (Figure 4A). However, plants infected with PSTVd-I:C42U/64U showed a slight increase in the expression of *PR1b1* mRNA from 3- to 5-wpi. The increase in *PR1b1* mRNA was validated by Northern blot assay (Figure S5A). Taken together, expression of *PR1b1* gene expression was positively correlated with the accumulation and pathogenesis of PSTVd and the tested mutants.

It has been demonstrated that the expression level of tomato gene *TCHS2* decreased in PSTVd-infected tomato plants after the manifestation of dwarfing symptoms [53,59]. To verify the effect of PSTVd mutants on the expression levels of tomato gene *TCHS2,* RT-qPCR was performed on total RNA extracted from tomato plants infected with PSTVd-I_wt_, PSTVd-D_wt_, PSTVd-I:C42U, PSTVd-I:64U and PSTVd-I:C42U/64U, respectively (Figure 4B). At 3-wpi, all the viroid inoculated plants exhibited a decrease in the expression levels of *TCHS2* mRNA. RT-qPCR data agreed with Northern blot assay (Figure S5B), that is, the expression of *TCHS2* mRNA is negatively correlated with the disease symptom expression during the onset of symptoms.

**Figure 5:**
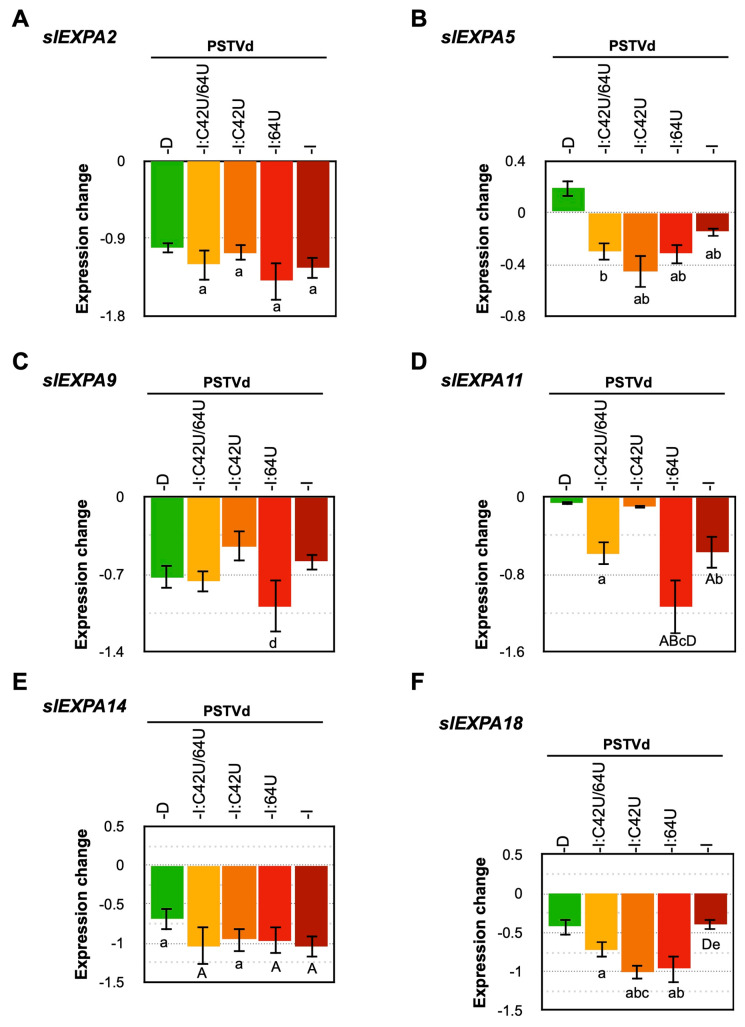
PSTVd infection differentially affects the expression of six expansin genes of tomato. Total RNA extracted from tomato plants inoculated with LMW-RNA of PSTVd-D_wt_, PSTVd-I_wt_ and mutants of PSTVd-I (PSTVd-I:C42U/64U, PSTVd-I:C42U, and PSTVd-I:64U) at 3-wpi were assayed by RT-qPCR to analyze the expression level of (**A**) *EXPA*2, (**B**) *EXPA*5, (**C**) *EXPA*9, (**D**) *EXPA*11, (**E**) *EXPA*14, and (**F**) *EXPA*18 mRNAs. The vertical axis indicates the relative expression level (Log2) of mRNAs. The data were normalized by the mean value of the mock-inoculated control at 1-wpi as 1.0. A *t*-test was performed with a *p*-value of 0.05. The plants that showed a difference < 0.05 and <0.01 are indicated by lower and capital letters on the top of the bars, respectively. In the figure, significant difference against the mock-inoculated control plant, PSTVd-D_wt_, PSTVd-I:C42U/64U, PSTVd-I:C42U, and PSTVd-I:64U is represented as a or A, b or B, c or C, d or D, and e or E, respectively. Mean values are based on three biological and three technical replicates.

The PSTVd mutant which induced flat-top symptoms in tomato plants exhibited a significant decrease in the expression of the *slEXPA2* gene [32]. Hence, the expression sites and patterns of those *slEXPA* genes were searched using the database CoNekT (https://conekt.sbs.ntu.edu.sg/). Those expressed in leaf and apical meristem tissues, specifically, *slEXPA2*, *slEXPA5*, *slEXPA9*, *slEXPA11*, *slEXPA14*, and *slEXPA18* genes, were analyzed by RT-qPCR. Data obtained at 3-wpi revealed that the expression of *slEXPA2*, *slEXPA9*, *slEXPA14*, and *slEXPA18* genes were significantly decreased in all plants infected with PSTVd (Figure 5 and Figure S6). Although the downregulation of *slEXPA5* and *slEXPA11* was observed in some PSTVd infected samples, the difference was noticeable. Taken together, these results showed that PSTVd infection downregulated almost all the *slEXPA* genes analyzed but no correlation was found between the level of downregulation and the pathogenicity of PSTVd and the mutants.

## 3. Discussion

PSTVd-D_wt_ was known to accumulate slowly and induce mild disease symptoms in tomato plants cv. Rutgers. Conversely, PSTVd-I_wt_ accumulates faster and prompts severe disease symptoms in the same host [62]. These two PSTVd isolates differ by nine nucleotides, six of which are located in the region of TL-P domains. Since the involvement of the TL-P domains in the pathogenicity of pospiviroids such as PSTVd, citrus exocortis viroid, tomato apical stunt viroid, or tomato planta macho viroid has been reported so far [67,68], the elucidation of the detailed molecular mechanism is particularly important. The involvement of the TL-P domains of PSTVd-D_wt_ in the expression of mild symptoms in infected tomato plants has been characterized in our previous report [69]; therefore, we have focused on the six nucleotides differences in these domains. To further understand the role of these six nucleotides in the pathogenicity and accumulation in more detail, 10 point-mutants of PSTVd (five point-mutants of each PSTVd-I_wt_ and PSTVd-D_wt_) were created by reciprocally exchanging the nucleotide at positions 42, 43, 64, 310, and 312 on PSTVd-I_wt_ and PSTVd-D_wt_.

Genetic stability analysis of these mutants in the infected tomato plants revealed that the mutations introduced at positions 42 in PSTVd-I (PSTVd-I:C42U) and 43 of PSTVd-D (PSTVd-D:C43U) were maintained stably, indicating nucleotides UU at positions 42 and 43 are stable (Table 1). Although PSTVd-D:C43U maintained mild pathogenicity similar to that of PSTVd-D_wt_, PSTVd-I:C42U resulted in attenuated symptom severity of PSTVd-I_wt_, comparable to that of PSTVd-D_wt_ (Figure 1). These results indicated that the importance of the nucleotide at position 42 in the pathogenicity, and also revealed that the combination of nucleotides 42 and 43 is important for genetic stability. When the combination of 42 and 43 is CU, as represented by PSTVd-I_wt_, it is stable and induces severe pathogenicity. On the other hand, as represented by PSTVd-D_wt_ and PSTVd-I:C42U, a combination of UC or UU is also stable but induces mild pathogenicity. Meanwhile, when the combination is CC, as represented by PSTVd-I:U43C or PSTVd-D:U42C, the mutant is unable to replicate stably thus inducing conversion from CC to CU. It should be noted that the resulted combination CU at position 42 and 43 is identical to that of PSTVd-I_wt_ and the resulted mutant of PSTVd-D:U42C accumulated faster and induced symptom slightly severer compared to the PSTVd-D_wt_ inoculated plants (Figure 1). These findings are in agreement with previous mutagenic studies where a PSTVd-D_wt_-derived mutant having CU at positions 42 and 43 (identical to PSTVd-I_wt_), induced slightly stronger symptoms compared to PSTVd-D_wt_ [42].

Accounting for all of the three PSTVd-I mutants and their comparison with PSTVd-I_wt_ and PSTVd-D_wt_ infected plants, results suggest that the nucleotide at position 42 has a significant effect on multiplication as well as accumulation, thus playing a key role in causing the mild symptoms of PSTVd-D_wt_. Although the insertion of a U nucleotide at position 63/64 attenuated the disease symptoms, its effect is less significant than that of nucleotide 42. For instance, vein necrosis was observed in PSTVd-I:64U infected plants but not PSTVd-I:C42U (Figure S3). Furthermore, when both nucleotides of PSTVd-I_wt_ at positions 42 and 64 were changed to PSTVd-D_wt_, the resulted mutant PSTVd-I:C42U/64U was shown to replicate stably. However, the multiplication was severely impaired, to levels lower than that of PSTVd-D_wt_ (Figure 2). This is a signature of the potential adverse effects caused by the multiplication of double mutations.

Regarding the possible effects of the mutations on the secondary structure, in silico computational analysis indicated that an insertion of 64U can induced conformational changes in the secondary structure of the P domain. Furthermore, analysis of the genetic stability of the mutants revealed that when a 64U was inserted into PSTVd-I_wt_, it was stably maintained, but when a 64U was deleted from PSTVd-D_wt_, the deletion was retained but accompanied another deletion of 310C in the paired lower strand of the P domain. Along with the drastic decrease in the accumulation in the double mutant PSTVd-I:C42U/64U, this result suggests the possible involvement of other mutations, such as those at positions 310 and 312 in the lower strand of P domain, in multiplication of PSTVd-D_wt_.

**Figure 6:**
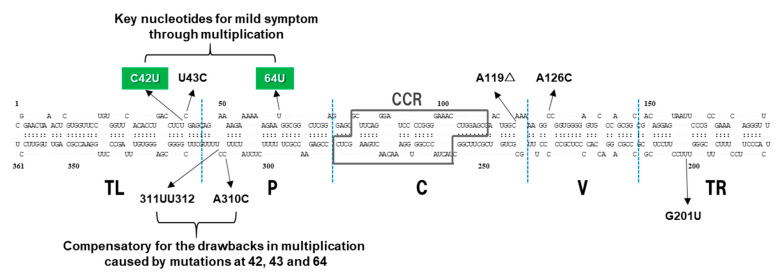
Proposed molecular mechanism of key nucleotides causing mild symptoms of PSTVd-D_wt_ in tomato. The structural/functional domains of PSTVd: Terminal left (TL), Pathogenicity (P), Central (C), Variable (V), and Terminal right (TR), are delimited by the vertical dotted lines and are named accordingly. Boxed sequences indicate central conserved region (CCR). Positions of nine mutations are indicated by arrows, and those in green box indicate key nucleotides identified in this experiment.

In the case of nucleotide 310 in the lower strand of the P domain, the mutations introduced in PSTVd-I:A310C and PSTVd-D:C310A were stably retained, but a C to A conversion in PSTVd-D:C310A incited a deletion of U from a tetra U sequence around the position 312 (Table 1), resulting in the formation of a triple U. In addition, the mutation introduced in PSTVd-D:312UUΔ (a UU deletion from a tetra U at positions 312–315 of PSTVd-D_wt_ resulting in UU) was stable. However, a UU insertion in PSTVd-I:312UU to form a tetra U caused a deletion of U from the sequence (Table 1) and resulted in the formation of a triple U which is similar to PSTVd-D:C310A. The result indicates that the combination of A in 310 and tetra U in 312–315 is incompatible in both PSTVd-I_wt_ and PSTVd-D_wt_ background, resulting in a covariation; i.e., a deletion in the tetra U sequence. It has been reported that a tetra U nucleotide at positions 312–315 characteristic to PSTVd-D_wt_ is a minor nucleotide sequence in nature. A major UU occupies 2/3 and a second major UUU occupies the remaining 1/3 of all isolates registered in the NCBI GenBank [62]. This indicates that a tetra U at nucleotide positions 312–315 in the lower strand of P in addition to UC at nucleotide positions 42–43 in the upper strand of TL are unique nucleotides that characterize PSTVd-D_wt_, a dahlia isolate. Although the infection assay indicated that nucleotide changes at 310 and 312 did not play a significant role in pathogenicity, these changes seemed to improve the disadvantages in PSTVd-D_wt_ multiplication caused by the two nucleotide changes in nucleotides 42–43 and 64 involved in mild pathogenesis. Therefore, it could be considered that nucleotides 310 and 312, in cooperation with nucleotides 42–43 and 64 in the paired strand, contribute to multiplication of PSTVd-D_wt_ through maintaining the local structure of the TL-P domains (Figure 6).

When attacked by the pathogens, plants activate innate immunity to defend themselves [70,71]. Numerous genes that indicate the activation of plant innate immunity, such as reactive oxygen species (ROS), pathogenesis-related (PR) proteins, calcium-dependent protein kinase, or salicylic acids, have been shown to increase in tomato plants infected with PSTVd [53,55,59]. These in turn cause severe symptoms such as necrosis by eliciting excessive defense responses [72]. Therefore, the tomato genes whose expression level is known to affect PSTVd infection was analyzed to verify the effect of PSTVd mutants on these genes in relation to pathogenicity. The expression level of *PR1b1* was extensively upregulated after PSTVd infection, by amounts that were highly correlated with the pathogenesis of the analyzed PSTVd mutants. The expression level in plants infected with PSTVd-I:C42U mutant was significantly lower than that in severe PSTVd-I_wt_ and PSTVd-I:64U mutants showing vein necrosis. That is, the mild pathogenesis of PSTVd-D_wt_ was thought to be partly attributable to the property that the isolate does not cause excessive responses in innate immunity in tomato because of its lower accumulation capacity. Chalcone synthase is a key enzyme in flavonoid biosynthesis pathways and catalyzes the synthesis of various secondary metabolites [73]. Previously, the tomato cultivars ‘Rutgers’ and ‘Heinz 1706’ infected with PSTVd-I, PSTVd-S23 (GenBank: X76846), or PSTVd-M (GenBank: X76844), exhibited stunted growth and leaf malformation as well as a significant reduction in the levels of expression of *TCHS2* gene [53,59]. Interestingly, PSTVd-I, PSTVd-S23, and PSTVd-M are all PSTVd isolates with a C at nucleotide position 42 and a deletion of U at position 64. Amongst tomato plants inoculated with PSTVd-I_wt_, the expression level of *TCHS2* gene declined to a level even lower than that of mock-inoculated control plants at 3-wpi when primary leaf symptoms began to appear and remained lower thereafter. In addition, in plants infected with attenuated PSTVd-I:C42U and PSTVd-I:64U, the expression levels of *TCHS2* gene also became lower than that of mock-inoculated control plants around 3- to 5-wpi in the late stage of infection. On the other hand, plants infected with mild PSTVd-D_wt_ and PSTVd-I:C42U/64U showed the same level of *TCHS2* gene expression as the mock-inoculated control plants even at 5-wpi. These observations indicated that there was an association between the severity of disease symptoms by PSTVd mutants and a decrease in the levels of *TCHS2* gene expression. Therefore, it was suggested that suppression of the expression of the *TCHS2* gene by PSTVd infection might have affected the onset of symptoms such as dwarfing and leaf malformation.

*Expansin* is a non-enzymatic protein found in the cell wall, with important roles in cell elongation [64]. In a previous study using the intermediate strain PSTVd^Int^ and its mutant PSTVd^Int^U257A with a U-to-A substitution at position 257, PSTVd^Int^U257A caused a more severe dwarfing effect than PSTVd^Int^, and it was reported that the expression of *slEXPA2* decreased significantly more than PSTVd^Int^ [32]. In this experiment, the expression levels of all six *slEXPA* genes were significantly down-regulated by the PSTVd except for *slEXPA5* in PSTVd-D_wt_ infected plants (Figure 5), regardless of their mutations or pathogenicity. Therefore, there was no direct relationship between the attenuation of PSTVd mutants and the suppression of *slEXPA* gene expression.

The six mutations found in the TL and P domains of PSTVd-D_wt_ and PSTVd-I_wt_ were characterized to study the molecular mechanisms underlying the property of PSTVd-D_wt_ to induce very mild symptoms in tomato plants. The mutagenic studies on two PSTVd variants suggested that mutations at nucleotides 42 and 64 were important; in particular, nucleotide 42 was the most critical nucleotide determining the attenuation of PSTVd-D_wt_ pathogenesis through multiplication and accumulation. Infection assays of mutants revealed a positive correlation between the pathogenicity and levels of increase in the expression of pathogenesis-related protein *PR1b1* gene and a decrease in the expression of chalcone synthase *TCHS2* gene. These results suggest that PSTV-D_wt_ does not elicit excessive host defense responses because of the lower accumulation due to nucleotide changes at 42 and 64, which is augmented by other changes at nucleotide positions 43, 310 and 311/312, consequently producing only mild symptoms.

## 4. Materials and Methods

### 4.1. Construction of PSTVd Dimeric cDNA Clones and Transcription of RNA Transcripts

Dimeric PSTVd-I_wt_ (GenBank accession no. M16826), PSTVd-D_wt_ (GenBank accession no. AB623143), and their mutants at positions 42, 43, 64, 310, and 311/312 (hereafter abbreviated as 312) were created by reciprocally exchanging individual nucleotide between PSTVd-I_wt_ and PSTVd-D_wt_ on the pBluescript II (SK–) (Stratagene, Japan) plasmid as described elsewhere [42,62,69]. The mutants are named as: PSTVd-I:C42U, PSTVd-I:U43C, PSTVd-I:64U, PSTVd-I:A310C, PSTVd-I:312UU, PSTVd-D:U42C, PSTVd-D:C43U, PSTVd-D:64Δ, PSTVd-D:C310A and, PSTVd-D:312UUΔ. An additional mutant, PSTVd-I:C42U/64U, a PSTVd-I-based mutant with two PSTVd-D-type mutations at positions 42 and 64, was constructed by inserting a chemically synthesized unit-length cDNA copy with *Bam*HI termini under the control of T7 RNA polymerase promoter sequence in pUC57 plasmid vector (GENEWIZ Japan Corp., Kawaguchi, Japan). Plasmid DNA (about 2 μg) containing the dimeric cDNA was digested with the *Asc*I or *Not*I restriction enzyme (Takara Bio Inc., Otsu, Shiga, Japan) at 37 °C overnight, and used for in vitro transcription at 37 °C for 2–4 h using T7 RNA polymerase (Invitrogen, Carlsbad, CA, USA) in a 20-μL reaction mixture according to the manufacturer’s instructions. The transcribed RNAs were recovered with ethanol precipitation, dissolved in 50 μL sterilized distilled water. Integrity of in vitro transcripts were examined by agarose gel electrophoresis followed by quantification using UV spectrophotometry [74].

### 4.2. Infection Assay to Analyze the Infectivity and Genetic Stability of Ten Types of PSTVd Mutants and Total RNA Extraction

Three seedlings of each indicator tomato plant (*Solanum lycopersicum*, cv. ‘Rutgers’) at the third true-leaf stage were inoculated with in vitro synthesized transcripts of PSTVd dimeric RNA in the first infection assay as described previously [62]. To further analyze the genetic stability of PSTVd mutants, the second passage of infection assay was conducted using native PSTVd RNAs obtained in the first infection assay. Inoculated plants were maintained in growth chamber for 2 months, with each day lasting 16 h at 25 °C at daylight intensity 3000–4000 lx (fluorescent, 40 W × 4), and night lasting 8 h at 22 °C.

For total RNA extraction, about 1 g of the upper uninoculated leaves were sampled at every week up to 5 weeks and homogenized in 5 mL of 2 × cetyltrimethylammonium bromide (CTAB) buffer [10 mL of 1 M Tris–HCl (pH 9.5), 46.6 mL of 3 M NaCl, 10 mL of 0.2 M disodium ethylenediaminetetraacetic acid (EDTA; pH 7.0), 2 g CTAB powder, 0.5 mL 2-mercaptoethanol, and 28.4 mL distilled water in 100 mL] [75] or TRIzol Reagent (Thermo Fisher Scientific, Waltham, MA, USA) according to the manufacturer’s instructions. Total RNA or low molecular weight (LMW) RNA obtained from the total RNA by fractionating 2 M LiCl-soluble nucleic acids and digestion of DNA by RQ1 *RNase*-free DNase I (Promega, Madison, WI, USA) [76], was used for the analyses of infectivity, accumulation, and genetic stability of mutants.

### 4.3. Infection Assay to Compare the Pathogenicity of Selected PSTVd Mutants

For the infection assay to compare the pathogenicity using three selected PSTVd mutants (PSTVd-I:C42U, PSTVd-I:64U, and PSTVd-I:C42U/64U) in comparison to the parental PSTVd-I_wt_ and PSTVd-D_wt_, LMW-RNA containing 100 pg of PSTVd RNA was dissolved in 20 µL of 50 mM sodium phosphate buffer (pH 7.0)—1 mg/mL bentonite for inoculation. Nine ‘Rutgers’ tomato seedlings per mutant were infected and incubated under the same conditions as above. They were later divided into three groups, and a leaf disk (taken from the topmost or the second topmost expanding or expanded leaf) of 1 cm in diameter per plant was collected every week until 5-wpi. Three leaf disks were combined in one tube (i.e., a total of three replicates per mutant) and used for total RNA extraction using TRIzol Reagent according to the manufacturer’s instruction.

### 4.4. Sequence Analysis of PSTVd Progeny

To analyze the genetic stability of the mutants, cDNA copies of the progenies were amplified using reverse transcriptase-polymerase chain reaction (RT-PCR). Total RNA extracts of about 100 ng/μL were reverse-transcribed using M-MuLV reverse transcriptase (Invitrogen, Carlsbad, CA, USA) in the presence of random hexamer primers (0.5 μM final concentration) in a 20 μL reaction at 37 °C for 60 min. Then, 2 μL of the RT solution was mixed with final 2 μM of each of the PSTVd-specific primer set; PS88M (5′-CCCTGAAGCGCTCCTCCGAG-3′) and PS89P (5′-ATCCCCGGGGAAACCTGGAGCGAAC-3′) [77], and PCR was performed using LA *Taq* polymerase (Takara Bio Inc. Shiga, Japan). The amplicons were ligated to a pGEM-T vector (Promega, Madison, WI, USA) and cloned in *Escherichia coli* DH5α JET competent cell (BioDynamics Laboratory Inc., Tokyo, Japan). DNA sequencing was performed either in-house with an ABI 3500 Genetic Analyzer (Applied Biosystems, Foster City, CA, USA) or at a commercial facility (Solgent Co., Seoul, Korea).

### 4.5. Secondary Structure Prediction of Mutants and Target Search for PSTVd-sRNA

The thermodynamically stable secondary structure of PSTVd mutants was calculated using mfold webserver [78]. Genes containing sequences that are highly complementary to PSTVd-derived small RNA (PSTVd-sRNA) were searched using psRNATarget:A Plant Small RNA Target Analysis Server [79].

### 4.6. Northern Blot Hybridization

The total RNA preparation (500 ng/μL) was denatured at 68 °C for 15 min and fractionated by electrophoresis at 100 V for 10 min on a 1.2% (*w*/*v*) agarose gel in 1× 3-(*N*-morpholino) propane sulfonic acid (MOPS) buffer. RNA was then transferred to a nylon membrane (Biodyne; Pall Corp., Port Washington, NY, USA) using a vacuum blotting apparatus (Vacuum Blotter Model 785; Bio-Rad Laboratories, Hercules, CA, USA) for 30–90 min at 5 inches mercury (Hg), UV-crosslinked at 120,000 µJ/cm^2^ in a UV crosslinker (UVP CX-2000; BM Equipment Co., Ltd., Tokyo, Japan), and hybridized with digoxigenin (DIG)-labeled cRNA probes as described previously [61]. DIG-labeled cRNA probes for tomato genes *EXPASIN* 2 (*slEXPA2*; DDBJ accession no., AF096776), and chalcone synthase (*TCHS2*; DDBJ, accession no., X55195) were prepared from recombinant plasmid DNAs containing a partial sequence of the gene in pBluescript II SK(–) under the control of either T7 or T3 RNA polymerase promoter constructed in this study. Hybridized signals were visualized using the ChemiDoc-XRS (Bio-Rad Laboratories, Hercules, CA, USA) imaging system and quantified using the Quantity One (version 4.6.2) software package.

### 4.7. Statistical Analysis

Assuming the data obtained are normally distributed, an F-test was performed to check whether the data are evenly distributed. The calculation was performed using Excel, and it was assumed that the variance is unequal when the *p* value was 0.05 or less. The data assumed to be homoscedastic were checked for significant differences using Student’s *t*-test. The data assumed to be unequal variances were checked for significant differences using Wilch’s *t*-test. A two-sided test was performed using Excel, and when the *p* value is 0.05 or less, it was judged that there is a significant difference.

### 4.8. RT-Quantitative PCR (RT-qPCR)

Total RNA preparation (5 μg) extracted using the TRIzol Reagent was treated with *RNase*-free *DNase* I (RQ1 DNase; Promega, Madison, WI, USA). According to the manufacturer’s instructions, cDNA was synthesized from 0.5 µg RNA using Superscript IV VILO (Invitrogen, Thermo Fisher Scientific, Tokyo, Japan) with random hexamers as primers. Quantitative PCR (qPCR) analysis was performed on the AriaMX real-time PCR system G8830A (Agilent Technologies, Santa Clara, CA, USA) using Brilliant III Ultra-Fast SYBR Green qPCR Master Mix (Agilent Technologies, Santa Clara, CA, USA). qPCR primers used for analysis are shown in Table S1. A preliminary test confirmed that the β-actin gene was most stably expressed regardless of the infection of PSTVd among the examined conditions under the present experimental conditions during the observation period, and thus was used as an internal standard. RT-qPCR results were normalized to the β-actin gene, and relative accumulation and transcript levels were calculated using the 2^−^^ΔΔC(t)^ method.

## Figures and Tables

**Table 1:** Summary of infectivity, genetic stability, accumulation, and pathogenicity of 10 potato spindle tuber viroid (PSTVd) mutants in tomato plant in the first and the second passages.

PSTVd Construct	Nucleotide Mutated					Northern-Blot Assay	Genetic Stability (4 wpi)		Systemic Accumulation	Pathogenicity
	42	43	64	310	312		1st Passage	2nd Passage		
PSTVd-I	C	U	-	A	-	+	N.T. *1	stable (13/13)	fast	severe
PSTVd-D	U	C	U	C	UU	+	N.T. *1	stable (10/10)	slow	mild
I-C42U	U	U	-	A	-	+	stable (11/11)	stable (11/11)	fast	mild
I-U43C	C	C	-	A	-	+	revert (2/13)	revert (10/10)	(fast) *2	(severe) *2
I-64U	C	U	U	A	-	+	stable (14/14)	stable (15/15)	fast	mild
I-A310C	C	U	-	C	-	+	stable (13/13)	stable (11/11)	fast	severe
I-312UU	C	U	-	A	UU	+	unstable (UU > U; 10/12)	unstable (UU > U; 12/12)	(fast) *2	(severe) *2
D-U42C	C	C	U	C	UU	+	covariation (C43U; 10/11)	covariation (C43U; 12/12)	(slow) *2	(mild) *2
D-C43U	U	U	U	C	UU	+	stable (12/12)	stable (11/11)	slow	mild
D-64Δ	U	C	-	C	UU	+	covariation (310CΔ; 5/12)	covariation (310CΔ; 10/10)	(slow) *2	(mild) *2
D-C310A	U	C	U	A	UU	+	covariation (312UΔ; 5/10)	covariation (312UΔ; 10/10)	(slow) *2	(mild) *2
D-312UUΔ	U	C	U	C	-	+	stable (12/12)	stable (10/10)	slow	mild

Nucleotides with blue background indicate PSTVd-I-type and those with green background indicate PSTVd-D-type. Numbers in parentheses indicate the number of cDNA clones which maintained stably or reverted to wild type or produced co-variation(s) per total sequence analyzed. *1. N.T. means not tested. *2. Since these mutants did not replicate stably, systemic accumulation and pathogenicity do not indicate the attributes of the original mutant itself.

## Supplementary Materials

Supplementary materials can be found at https://www.mdpi.com/1422-0067/21/19/7352/s1. Table S1: Oligonucleotide primers used in the present study. Figure S1: Northern blot hybridization to evaluate the accumulation of native PSTVd mutants. Figure S2: (A) RT-PCR, and (B) Northern blot hybridization analysis for the accumulation of PSTVd-I:C42U/64U in tomato plants. Figure S3: Tomato plants inoculated with PSTVd-I_wt_ and PSTVd-I:64U exhibited severe vein necrosis in the middle of leaves at 5-wpi. Figure S4: RT-qPCR analysis for the accumulation of PSTVd-D_wt_, PSTVd-I:C42U, and PSTVd-I_wt_. Figure S5: Northern blot hybridization for the accumulation of (A) *PR1b1*, and (B) *TCHS2* mRNA. Figure S6: Northern blot hybridization for the accumulation of *EXPA*2 mRNA.
