# Supplementary material for: Identification and Molecular Mechanisms of Key Nucleotides Causing Attenuation in Pathogenicity of Dahlia Isolate of Potato Spindle Tuber Viroid
Source: Int J Mol Sci. 2020 Oct 5;21(19):7352. doi: 10.3390/ijms21197352 (PMC7583970; doi:10.3390/ijms21197352)
Supplement: Supplementary file 1 [file ijms-21-07352-s001.pdf]

Table S1: Oligo nucleotide primers used in the present study

Name	sequence (5'---3')	References
PSTVd-231F<br>PSTVd-296R	GCCCCCTTTGCGCTGT<br>AAGCGGTTCTCGGGAGCTT	[1]
SlEXPA2-F<br>SlEXPA2-R	TTGCACCCGCTTAGGCCTATTAGT<br>ACCCTCAAATCCATAAATGGCCGC	[2]
SlEXPA5-F<br>SlEXPA5-R	GGCAGAATAACGCTTACCTTAACGGC<br>GACCAAGAACTAGGAGCTGCATTG	
SlEXPA9-F<br>SlEXPA9-R	CCCATCACACTGGCAATTTGGTCA<br>TCAGCTCTTCTACATGCACCACCA	
SlEXPA11-F<br>SlEXPA11-R	ATTTATACGTGTGATAGGCAGCGGCG<br>TGTTTCCAGCACCTTCGGACTAGA	
SlEXPA14-F<br>SlEXPA14-R	AGCTGGCATTGTCCCTGTCATCTA<br>TCTCCTGCACCTCCAACGTTTGTT	
SlEXPA18-F<br>SlEXPA18-R	TGGGAAGGGATGAAGCGTAGATGA<br>AGTCCTAATAGAAGCTGCGGGCTA	
tchs2-1152P<br>tchs2-1255M	CTCATCCAAAGAAGGGCTTAGTACC<br>CACTATGGAGCACAACAGTCTCAAC	This work
β-actin F<br>β-actin R	GAGGACAGGATGCTCCTCAG<br>AGACGCCTATGTGGGAGATG	[3]
PSTV-88M<br>PSTVd-89P	CCCTGAAGCGCTCCTCCGAG<br>ATCCCCGGGGAAACCTGGAGCGAAC	[4]

Reference:

1. Adkar-Purushothama, C.R.; Brosseau, C.; Giguère, T.; Sano, T.; Moffett, P.; Perreault, J.-P. Small RNA Derived from the Virulence Modulating Region of the Potato spindle tuber viroid Silences callose synthase Genes of Tomato Plants. *Plant Cell* **2015**, *27*, 2178–2194, doi:10.1105/tpc.15.00523.
2. Lu, Y.; Liu, L.; Wang, X.; Han, Z.; Ouyang, B.; Zhang, J.; Li, H. Genome-wide identification and expression analysis of the expansin gene family in tomato. *Mol. Genet. Genomics* **2016**, doi:10.1007/s00438-015-1133-4.
3. Wu, W.; Ding, Y.; Wei, W.; Davis, R.E.; Lee, I.-M.; Hammond, R.W.; Zhao, Y. Salicylic acid-mediated elicitation of tomato defence against infection by potato purple top phytoplasma. *Ann. Appl. Biol.* **2012**, *161*, 36–45, doi:10.1111/j.1744-7348.2012.00550.x.
4. Tsushima, D.; Tsushima, T.; Sano, T. Molecular dissection of a dahlia isolate of potato spindle tuber viroid inciting a mild symptoms in tomato. *Virus Res.* **2016**, *214*, 11–18, doi:10.1016/j.virusres.2015.12.018.

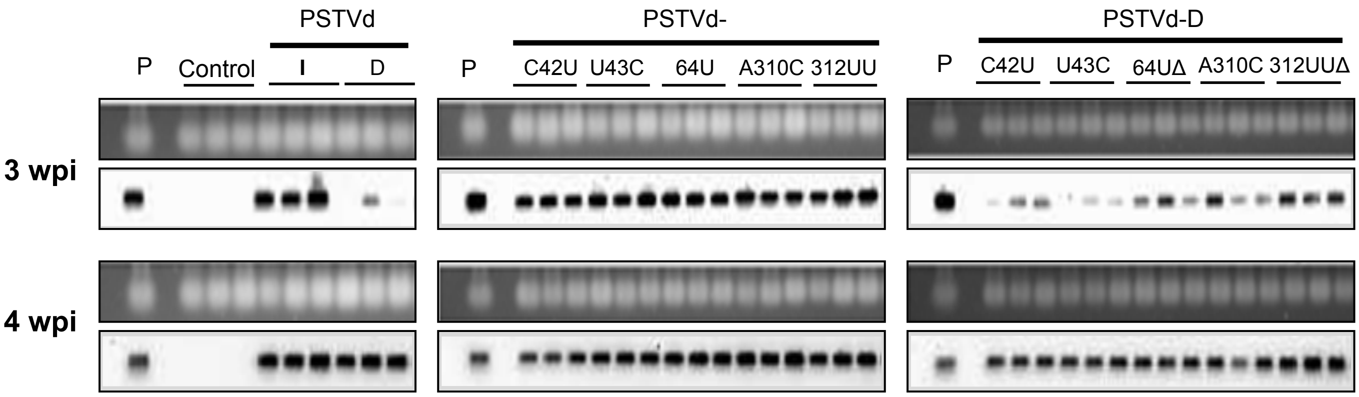

Figure S1: Northern blot hybridization to evaluate the accumulation of native PSTVd mutants.

Total RNA extracted from tomato plants inoculated with native PSTVd mutants were analyzed by Northern blot assay using DIG-labelled PSTVd specific probes at 3-, and 4-wpi. In the figure, P denotes, positive control; control, mock-inoculated plants.

Figure S1 (Kitabayashi et al.,)

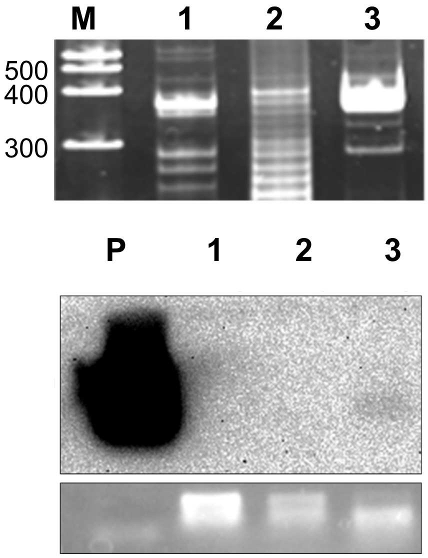

Figure S2: (A) RT-PCR, and (B) Northern blot hybridization analysis for the accumulation of PSTVd-I:C42U/64U in tomato plants.

In the figure, M denotes 100-bp ladder; P indicates positive control.

Figure S2 (Kitabayashi et al.,)

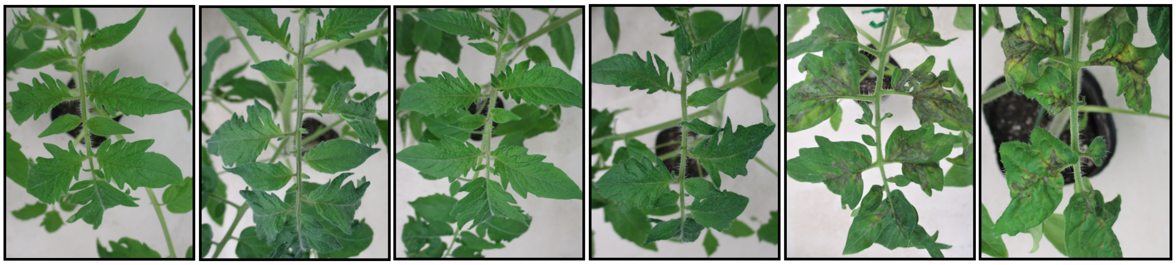

Control PSTVd-D PSTVd-I:C42/64U PSTVd-I:C42U PSTVd-I:64U PSTVd-I

Figure S3: Tomato plants inoculated with PSTVd-I and PSTVd-I:64U exhibited severe vein necrosis on the middle of leaves at 5 wpi.

Figure S3 (Kitabayashi et al.,)

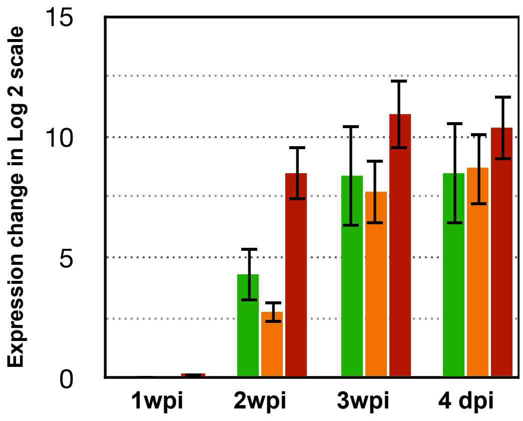

Figure S4: RT-qPCR analysis for the accumulation of PSTVd-D_{wt}, PSTVd-I:C42U, and PSTVd-I_{wt}.

Total RNA extracted from tomato plants inoculated with LMW-RNA of PSTVd-D_{wt}, PSTVd-I_{wt} and PSTVd-I:C42U were assayed by RT-qPCR to analyze the accumulation of viroid RNA. The expression of the viroid RNA is presented in a log2 scale. In the figure, the green bar represents the PSTVd-D_{wt} inoculated plants, while, the orange and the red bars indicate the PSTVd-I:C42U and PSTVd-I_{wt} inoculated plants, respectively. Data represent the mean of three independent experiments, each performed in triplicate. Error bars indicate SD.

Figure S4 (Kitabayashi et al.,)

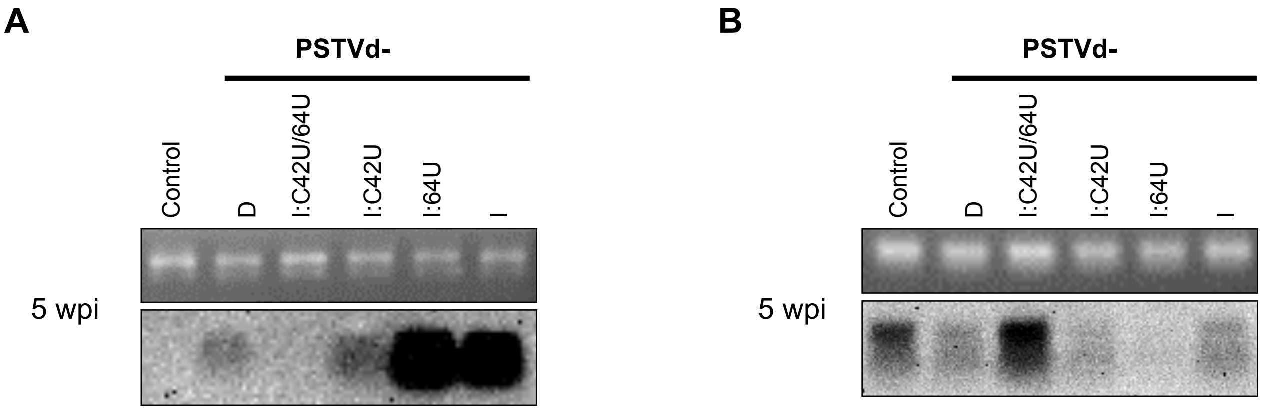

Figure S5: Northern gel blot assay for the accumulation of (A) *slPR1b1*, and (B) *TCHS2* mRNA

Total RNA extracted from tomato plants inoculated with LMW-RNA of PSTVd-D_{wt}, PSTVd-I_{wt}, and mutants of PSTVd-I (PSTVd-I:C42U/64U, PSTVd-I:C42U, and PSTVd-I:64U) at 5-wpi were assayed by Northern gel blot to analyze the expression level of (**A**) *PR1b1* and (B) *TCHS2* mRNAs.

Figure S5 (Kitabayashi et al.,)

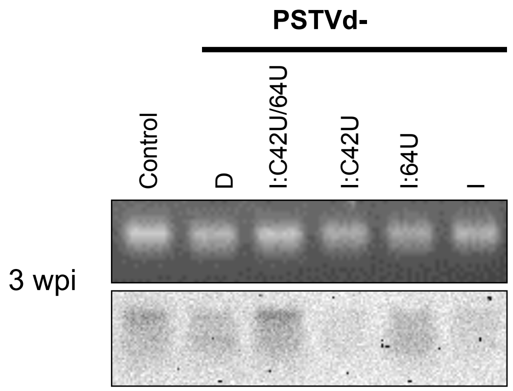

Figure S6: Northern gel blot assay for the accumulation of *EXPA*2 mRNA.

Total RNA extracted from tomato plants inoculated with LMW-RNA of PSTVd-D_{wt}, PSTVd-I_{wt} and mutants of PSTVd-I (PSTVd-I:C42U/64U, PSTVd-I:C42U, and PSTVd-I:64U) at 5-wpi were assayed by Northern gel blot to analyze the expression level of *EXPA2* mRNA.

Figure S6 (Kitabayashi et al.,)
